# Role and Function of O-GlcNAcylation in Cancer

**DOI:** 10.3390/cancers13215365

**Published:** 2021-10-26

**Authors:** Jii Bum Lee, Kyoung-Ho Pyo, Hye Ryun Kim

**Affiliations:** 1 Division of Hemato-Oncology, Wonju Severance Christian Hospital, Yonsei University Wonju College of Medicine, Wonju 26426, Korea; jiibumlee@yonsei.ac.kr; 2 Division of Medical Oncology, Department of Internal Medicine, Yonsei Cancer Center, Yonsei University College of Medicine, Seoul 06273, Korea; 3 Department of Medical Science, Yonsei University College of Medicine, Seoul 06273, Korea

**Keywords:** O-GlcNAcylation, O-GlcNAc transferase, O-GlcNAcase, cellular stress, cancer, immune surveillance

## Abstract

**Simple Summary:**

Despite the rapid advancement in immunotherapy and targeted agents, many patients diagnosed with cancer have poor prognosis with dismal overall survival. One of the key hallmarks of cancer is the ability of cancer cells to reprogram their energy metabolism. O-GlcNAcylation is an emerging potential mechanism for cancer cells to induce proliferation and progression of tumor cells and resistance to chemotherapy. This review summarizes the mechanism behind O-GlcNAcylation and discusses the role of O-GlcNAcylation, including its function with receptor tyrosine kinase and chemo-resistance in cancer, and immune response to cancer and as a prognostic factor. Further pre-clinical studies on O-GlcNAcylation are warranted to assess the clinical efficacy of agents targeting O-GlcNAcylation.

**Abstract:**

Cancer cells are able to reprogram their glucose metabolism and retain energy via glycolysis even under aerobic conditions. They activate the hexosamine biosynthetic pathway (HBP), and the complex interplay of O-linked N-acetylglucosaminylation (O-GlcNAcylation) via deprivation of nutrients or increase in cellular stress results in the proliferation, progression, and metastasis of cancer cells. Notably, cancer is one of the emerging diseases associated with O-GlcNAcylation. In this review, we summarize studies that delineate the role of O-GlcNAcylation in cancer, including its modulation in metastasis, function with receptor tyrosine kinases, and resistance to chemotherapeutic agents, such as cisplatin. In addition, we discuss the function of O-GlcNAcylation in eliciting immune responses associated with immune surveillance in the tumor microenvironment. O-GlcNAcylation is increasingly accepted as one of the key players involved in the activation and differentiation of T cells and macrophages. Finally, we discuss the prognostic role of O-GlcNAcylation and potential therapeutic agents such as O-linked β-N-acetylglucosamine-transferase inhibitors, which may help overcome the resistance mechanism associated with the reprogramming of glucose metabolism.

## 1. Introduction

One of the pivotal hallmarks of cancer is reprogramming energy metabolism in cancer cells [1]. Normally, cells process glucose under aerobic conditions and favor glycolysis under anaerobic conditions. However, cancer cells differ and reprogram their glucose metabolism to resourcing their energy metabolism mainly to glycolysis [2]. Cancer cells prefer glycolysis instead of oxidative phosphorylation, despite the presence of high oxygen; the effect known as the Warburg effect [3]. Although mitochondrial oxidative phosphorylation produces ATP with approximately 18-fold higher efficiency than aerobic glycolysis, cancer cells manage to compensate energy metabolism by the HBP [4,5]. Thus, approximately 3–5% of glucose is diverted to the HBP, whereas most of the glucose molecules are metabolized through glycolysis [4].

Notably, one of the emerging mechanisms of cancer metabolism behind this complex interplay of glucose metabolism is O-GlcNAcylation, a non-canonical glycosylation that is activated as a response to stimuli such as nutrient deprivation and cellular stress [6]. Since the first discovery of O-GlcNAcylation in 1984, several hypothesis and key concepts have resulted in the understanding of how cellular O-GlcNAcylation is controlled by nutrients and hormones [7]. The key nutrients and metabolic intermediates, such as glucose, amino acid, fatty acid, and nucleotide, are utilized and converted by the HBP to generate the uridine diphosphate GlcNAC (UDP-GlcNAc), a donor substrate for O-GlcNacylation [5]. Subsequently, UDP-GlcNAc transfers O-linked-β-N-acetylglucosamine (O-GlcNAc) to the enzyme O-GlcNAc transferase (OGT), which attaches O-GlcNAc moieties to the serine and/or threonine residues of substrate proteins, including cytoplasmic, nuclear and mitochondrial proteins [8]. This process results in post-translational modification (PTM) of the substrate proteins known as called O-GlcNAcylation [9]. O-GlcNAcase (OGA) reverses the process by catalyzing the hydrolysis done by the OGT [10]. O-GlcNAcylation differs from other PTMs and is strictly controlled primarily by OGT and OGA. This single pair of enzymes recognize hundreds of protein substrates necessary for O-GlcNac homeostasis [9].

O-GlcNAcylation affects many diseases, including diabetes, diabetic nephropathy, and neurodegenerative disease such as Alzheimer disease [11,12,13,14,15]. In recent years, several studies have addressed the role of protein O-GlcNAcylation in various types of cancer, including the impact of O-GlcNAcylation in proliferation, angiogenesis, and metastasis of cancer cells [16,17]. This review summarizes the general mechanism of O-GlcNAcylation, including substrate recognition by OGT and OGA and functions of cellular O-GlcNAcylation. Finally, we discuss the role of O-GlcNAcylation from the perspectives of cancer, including metastasis, receptor tyrosine kinases (RTKs), resistance to chemotherapy, prognostic marker, tumor microenvironment, and the potential targeting of cellular O-GlcNAcylation as cancer therapeutics.

## 2. O-GlcNAcylation

### 2.1. Substrate Recognition by OGT and OGA

**Figure 1:**
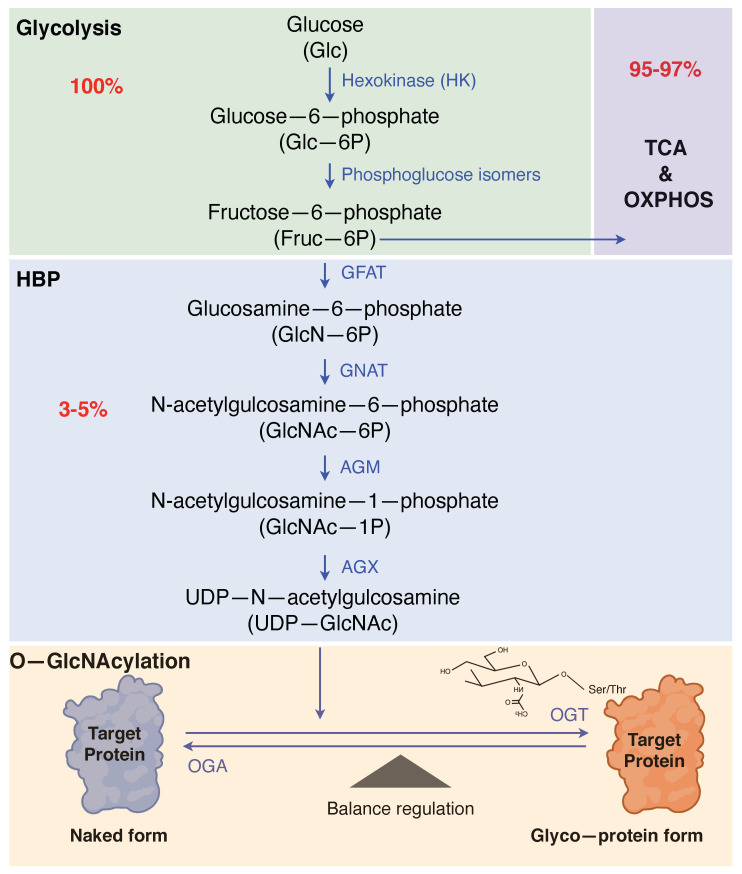
Hexosamine biosynthetic pathway and protein O-GlcNAcylation. Cancer cells compensate for energy metabolism by the hexosamine biosynthetic pathway. While most of the glucose is metabolized through glycolysis, approximately 3–5% of glucose enters the HBP. Glutamine-fructose-6-phosphate aminotransferase, the rate-limiting enzyme of the HBP, converts fructose-6-phosphate into glucosamine-6-phosphate. Subsequently, Glucosamine-6-P is acetylated and uridylation of Glucosamine-1-phosphate generates UDP-N-acetylglucosamine. UDP-GlcNAc acts a substrate, and O-GlcNAc-transferase and O-GlcNAcase add and remove GlcNAc to serine or threonine residues of target proteins, respectively. TCA, Tricarboxylic acid cycle; OXPHOS, Oxidative phosphorylation; HBP, hexosamine biosynthetic pathway GFAT1, hexosamine biosynthetic pathway; GNAT, N-acetyltransferase; AGM, N-acetylphosphoglucosamine mutase; AGX, UDP-N-acetylhexosamine pyrophosphorylase; OGA, O-GlcNAcase; OGT, O-GlcNAc-transferase.

Contrary to other PTMs that are regulated by diverse enzymes, O-GlcNAcylation is controlled by a single pair of enzymes, OGT and OGA, which recognize hundreds of protein substrates [6,8] (Figure 1). O-GlcNAc, is the master regulator of detained intron (DI) splicing, and regulates gene expression by controlling and splicing the DI in OGT and OGA [18]. Thus, the balance between OGT and OGA level is maintained in an O-GlcNAc-dependent manner. OGT has three isoforms: nucleocytoplasmic (ncOGT), mitochondrial (mOGT), and short OGT (sOGT). These isoforms differ in their subcellular locations and number of amino-terminal tetratricopeptide repeats (TPRs), and thereby differ in length. While ncOGT and sOGT reside in the cytoplasm and nucleus, mOGT is present in the mitochondria. The unique TPR domain length and locations of these isoforms enable targeting various subsets of proteome [19]. Substrates are specific to the isoforms of OGT, and the three isoforms are expressed differently [20,21]. For instance, mOGT is expressed transiently and in much lower than ncOGT, due to its susceptibility to the cellular glucose level [22,23]. The exact mechanisms for substrate recognition by OGT are yet to be clarified; however, the adaptor protein hypothesis and non-specific O-GlcNAcylation are recognized as the plausible mechanisms [24,25,26,27].

Under specific condition such as glucose deprivation and fasting, OGT substrate recognition may be mediated by proteins, including p38 mitogen-activated protein kinase (MAPK), host cell factor 1 (HCF1), and OGA, which act as adaptor proteins in their receptive substrates of neurofilament H (NFH), peroxisome proliferator-activated receptor-gamma coactivator (PGC)-1alpha (PGC1α), and pyruvate kinase M2 (PKM2), respectively [24,25,26]. Thus, O-GlcNAcylation is controlled by OGT via adaptor proteins in a context-dependent manner, similar to the ubiquitylation system in which E3 ubiquitin ligases act as adaptor proteins to the E2 ubiquitin-conjugating enzymes [28].

Non-specific O-GlcNAcylation hypothesis stems from the OGT to O-GlcNAcylate proteins in flexible regions, which include loops and termini that bind to the active site, thereby exposing the amide backbone [27]. The preferential modification of substrates that contain the flexible regions by OGA enables the modification of proteins without recognition of any specific sequences or structures. Most mature proteins have a limited number of flexible elements which prevent nonspecific O-GlcNAcylation in normal physiological conditions [29]. During cellular stress, however, non-specific O-GlcNAcylation takes place in unstructured regions of unfolded proteins, facilitating their folding and inhibiting degradation [25,30,31,32]. For instance, O-GlcNAcylation of unstructured polypeptides, including nascent specificity protein 1 (SP1) and nucleoporin 62 (NUP62) polypeptides, inhibits premature ubiquitin-mediated degradation, and thereby maintains protein homeostasis [33].

The two isoforms of OGA, nucleocytoplasmic isoform and short isoform, differ from each other; with the former having both an N-terminal O-GlcNAc hydrolase domain and C-terminal histone acetyltransferase-like (HAT-like), and the latter lacking the HAT-like domain [13,34]. Currently, information related to the mechanism behind the substrate recognition by OGA is limited due to the incomplete identification of the crystal structure of human OGA [35,36,37]. Bacterial glycosidases, such as those of *Clostridium perfringenes* and *Bacteroides thetaiotaomicron,* have shown structural similarities by avoiding contact with the peptide chains and instead binding to diverse substrates via interaction of the peptide backbone and sugar moieties [38,39,40]. Further elucidations of structural features of OGA may identify the mechanism behind the recognition of substrate by OGA.

### 2.2. Functions of O-GlcNAcylation

**Figure 2:**
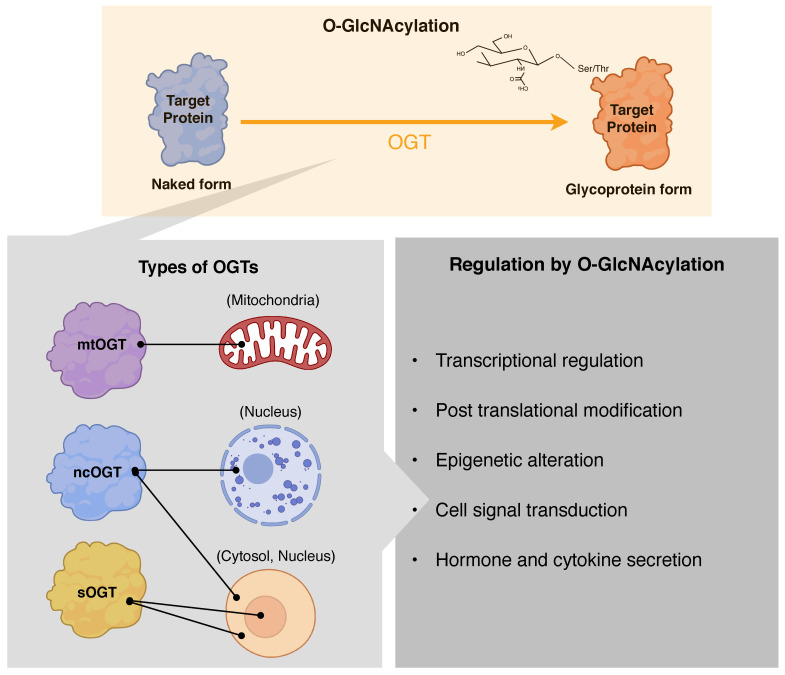
The isoforms of O-GlcNAc transferase (OGT) and the role of O-GlcNAcylation. OGT has three isoforms: nucleocytoplasmic OGT (ncOGT), mitochondrial (mOGT), and short OGT (sOGT). O-GlcNAcylation is involved in transcriptional regulation, post-translational modification, epigenetic alteration, cell signal transduction, and hormone and cytokine secretion.

Protein O-GlcNAcylation by the complex coordination of OGT and OGA results in regulation of transcription, epigenetic programs, temporal regulation of cell signaling dynamics, and nutrient and hormonal regulation (Figure 2) [41,42,43,44,45,46,47,48]. Both OGT and OGA are O-GlcNAcylated and autoregulated at post-translation level, and cellular stress, such deficient or excess nutrients, disrupts the regulation of cellular O-GcNAc homeostasis [9,11,13,49]. O-GlcNAcylation orchestrates translocation and DNA binding of transcriptional factors such as SP1, RNA polymerase II, and nuclear factor kappa-light-chain-enhancer of activated B cells (NF-κB) in a context-dependent manner [41,43,50]. Proteins such as HCF1 and Ten-eleven translocation (TET), involved in histone modification and DNA methylation, respectively, interact with OGT [34,43,44,51]. OGT regulation is associated with the temporal regulation of insulin signaling dynamics [45]. O-GlcNAcylation negatively regulates insulin signaling when phosphatidylinositol-3,4,5-trisphosphate, the key regulator of insulin signal transduction, recruits OGT from the cytoplasm to the membrane [48,52]. OGT then O-GlcNAcylates and negatively regulates insulin signaling pathway.

In addition to the diverse roles O-GlcNAcylation in the fundamental cellular process, its most crucial role is functioning as a nutrient sensor [53]. Previously, increased cellular O-GlcNAcylation was positively associated with the availability of nutrients in response to the influx through the HBP. In vitro and in vivo studies have shown hyperglycemia results in hyper O-GlcNAcylation, thereby supporting the view that HBP flux is the key determinant of an increase in O-GlcNAcylation [46,47]. Recently, evidence has shifted from a positive correlation to variations according to substrate-by-substrate basis [25]. Depending on the levels of nutrients available, OGT and its adaptor proteins modulate the levels of protein O-GlcNAcylation [24,25,26]. During nutrient deprivation, there is an increase in cellular O-GlcNAcylation, even though UDP-GlcNAc levels are decreased [24,46,47]. The upregulation of OGT expression and the high affinity of OGT for the abundant unfolded proteins during nutrient stress may potentially explain the increase in cellular O-GlcNAcylation despite the decreased level of UDP-GlcNAc. Thus, the complex interplay and harmony of OGT, OGA, and their proteins and substrates result in regulation of cellular protein O-GlcNAcylation.

## 3. O-GlcNAcylation and Cancer

### 3.1. O-GlcNAcylation and Metastasis

Several cancers, notably breast, colon, pancreas, liver, and lung cancers, have been associated with elevated O-GlcNAcylation [54,55]. The Cancer Genome Atlas datasets show the aberrant levels of OGT in both adenocarcinoma and squamous cell carcinoma of lung [56]. This results in invasion, metastasis, and angiogenesis of lung cancer cells by activating transcription factors such as Notch receptor 1 (Notch1) and nuclear factor erythroid 2-related factor (Nrf2) [57,58]. Notch-dependent metastasis is potentially modulated via O-GlcNAcylation [57,59].

**Figure 3:**
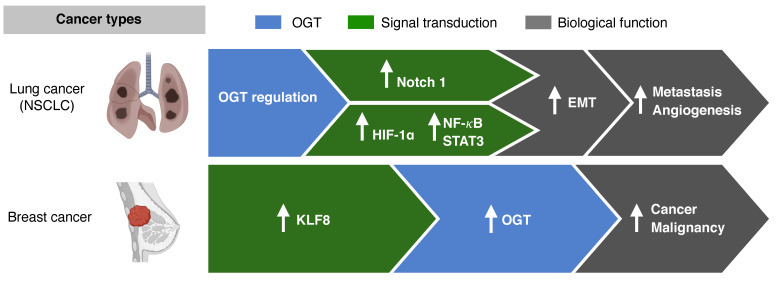
O-GlcNAcylation and metastasis. In non-small cell lung cancer (NSCLC), the epithelial mesenchymal transition (EMT) and transcriptional factors such as hypoxia-inducible factor-1α (HIF) and nuclear factor kappa-light-chain-enhancer of activated B cells (NF-κB) are mediated via O-GlcNAcylation. In breast cancer, the activation of Krüppel-like factor 8 (KLF8) results in OGT activation.

In NSCLC, O-GlcNAcylation mediates and sustains the epithelial mesenchymal transition (EMT) [60]. EMT markers, such as E-cadherin and vimentin, are suppressed when the O-GlcNAcylation levels are high (Figure 3) [61,62]. Cancer cells that undergo EMT have more aggressive and invasive features due to their ability to migrate [63]. Other transcriptional factors, such as hypoxia-inducible factor(HIF) -1α, nuclear factor kappa-light-chain-enhancer of activated B cells (NF-κB), and signal transducer and activator of transcription 3 (STAT3), are also activated via O-GlcNAcylation, resulting in cancer invasion and metastasis in cancers such as NSCLC, cervical cancer, and head and neck cancer [59,64,65].

In vitro and in vivo studies on breast cancer cell lines have shown that O-GlcNAc transferase regulates cancer stem-like potential [54]. Krüppel-like factor 8 (KLF8) acts as a novel regulator in mammosphere formation, and activation of KLF8 resulted in activation of OGT in xenograft tumors in vivo. Breast cancers with KLF8 showed worse OS than breast cancers without KLF8 expression. The role of OGT in the regulation of cancer-stemness and tumor metastasis, as seen in breast cancer, may potentially be targeted to overcome resistance to chemotherapy.

### 3.2. O-GlcNAcylation and Receptor Tyrosine Kinase

Activation of downstream pathways of RTK, such as KRAS and epidermal growth factor receptor (EGFR), is associated with an increased glucose flux via the HBP pathway [66,67]. Approximately 90% of the pancreatic ductal adenocarcinoma (PDAC) is associated with KRAS [68]. Increased cellular O-GlcNAcylation initiates reduction in ribonucleotide reductase activity and dNTP pools, resulting in genomic alterations, including *KRAS* mutations in PDAC [66]. Similarly, O-GlcNAcylation is associated with tumorigenesis in *KRAS*-mutant lung cancer [67]. In mouse models, the upregulation and downregulation of O-GlcNAcylation significantly accelerated and delayed *Kras^G12D^* lung tumorigenesis, respectively. *EGFR* mutations are also crucial in NSCLC, because more than 60% of patients diagnosed with NSCLC harbor *EGFR* mutations, and treatment with tyrosine kinase inhibitors (TKIs) is preferred over chemotherapy or immunotherapy for these subset of patients [69,70]. Contrary to the cell lines of cervical adenocarcinoma, the cell lines of lung adenocarcinoma are associated with EGFR O-GlcNAcylation in the serine and/or threonine residue(s) [71]. The PTM of EGFR via O-GlcNAcylation may possibly be tumor-specific, and warrants further exploration.

### 3.3. O-GlcNAcylation and Resistance to Chemotherapy

In lung carcinoma cells, hyper-O-GlcNAcylation is associated with cisplatin resistance via the regulation of either p53 or c-Myc [72]. O-GlcNAcylation of p53 and cMyc results in p53 instability due to ubiquitin-mediated proteasomal degradation and inhibition of c-Myc ubiquitination and degradation, respectively. Recent in vitro and in vivo studies also showed that protein O-GlcNac modification of p53 or c-Myc affects the anti-tumor activity of cisplatin in NSCLC cell lines [73]. Treatment with cisplatin increased the activities of OGT and OGA and decreased the activity of AMP-activated protein kinase. However, inhibition of OGT and OGA by altering O-GlcNAc levels did not result in an increased sensitivity of cisplatin in lung cancer cells.

In many types of cancers, cisplatin is the key chemotherapeutic in both the adjuvant and palliative settings [74]. Identifying the potential mechanism behind cisplatin resistance is an unmet need for patients with cancers, as most of the patients experience disease progression with chemotherapeutic agents [75]. In other cancer cell lines, such as hepatocellular carcinoma, inhibition of O-GlcNAc transferase resulted in enhancement of apoptosis by doxorubicin [76]. Further studies are warranted to elucidate the role of O-GlcNAcylation in the resistance to chemotherapeutic agents used in cancers such as navelbine, pemetrexed, carboplatin, and docetaxel, as well as potential therapeutic agents targeting hyper-O-GlcNAcylation.

### 3.4. O-GlcNAcylation as Prognostic Marker

Several studies have shown that cancers, including prostate and colorectal cancers, harboring hyper-O-GlcNAcylation are associated with worse prognosis [77,78]. In squamous cell lung cancer, O-GlcNAcylation and increased OGT levels were observed in lung cancer cells compared with the adjacent lung tissue [78]. The expression of OGT in patients with stages II, III, and IV lung adenocarcinomas was higher than that in patients with stage I lung adenocarcinoma [79]. In this study, stage I patients with high OGT expression had shorter recurrence-free survival (RFS) and poor OS. Multivariate analysis revealed that high OGT was a prognostic factor for both RFS and OS, indicating OGT as a potential biomarker in early-stage lung adenocarcinoma. The clinical significance of O-GlcNAcylation is yet to be determined with larger prospective cohorts and validation studies.

## 4. O-GlcNAcylation and Immune Responses in Cancer

### 4.1. Overview of Immune System and O-GlcNAcylation

**Figure 4:**
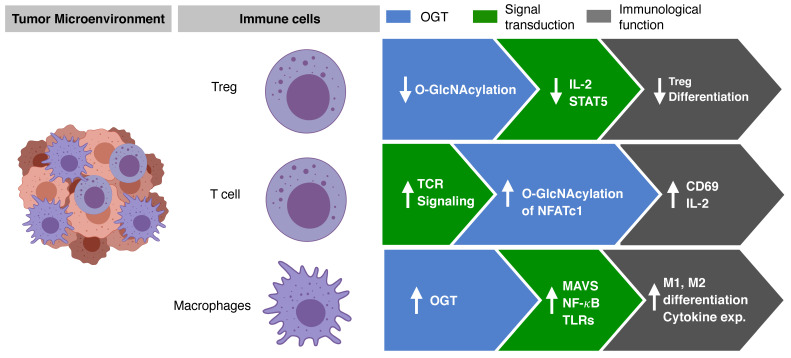
O-GlcNAcylation regulates immune cells activation and differentiation. O-GlcNAcylation is associated with the immune surveillance in tumor microenvironment. O-GlcNAcylation regulates the activation and differentiation of T cells and macrophages, thereby exerting signal transduction and immune surveillance. T reg, regulatory T cell; IL-2, interleukin-2; STAT5, signal transducer and activator of transcription 5; TCR, T-cell receptor; NFATc1, nuclear factor of activated T cells 1; MAVS, mitochondrial antiviral-signaling protein; NF-κB, nuclear factor kappa-light-chain-enhancer of activated B cells; TLRs, Toll-like receptors; exp., expression.

O-GlcNAcylation is highly related to the immune surveillance in the tumor microenvironment (Figure 4). The metabolic shift in immune cells affects T-cell activation and differentiation. Increased amounts of energy metabolites, such as glucose and amino acids, are required for T cells. Specifically, glutamine uptake is essential in activated T cells. Initially, glutamine is converted to a source of oxaloacetate in the tricarboxylic acid cycle (TCA) cycle. The acetyl-CoA is generated by the metabolism of TCA cycle, which allows for greater fatty acid synthesis. The produce of this metabolic pathway serves as a substrate in the HBP [80]. The role and function of O-GlcNAcylation for the two important immune cells constituting the tumor microenvironment have been studied. The effects of O-GlcNAcylation in the tumor microenvironment include dealing with the differentiation and signaling mechanisms of T cells and differentiation and activation of macrophages.

### 4.2. T Cell Activation and Differentiation Regulated by O-GlcNAcylation

In the thymus, lymphocyte development and activation were observed with increased O-GlcNAc level [81]. This finding suggested that the modification of O-GlcNAc occurred during the early stages of T lymphocyte activation. In addition, the OGT knockdown experiment showed an impairment of interleukin (IL)-2 production in the T cells. Furthermore, ZAP70, SHIP1, and LCK were identified as substrates of O-GlcNAc that regulate TCR signaling [82].

Helper T cells (Th cells) differentiate into several types of cells by the cytokines or surface ligands expressed in antigen-presenting cells or adjacent immune cells. Th cells comprise Th1, Th2, Th17, and regulatory T cells (Treg), which are activated in an abnormal environment, such as an infection or tumor. Finally, differentiated Th in the form of cytokines exhibit anti-tumor and anti-infection effects. However, differentiation of Th can also reduce the anti-tumor effect and damage the host due to excessive immune response. In general, anticancer immune responses are regulated by cellular immunity, and in particular, Th1-type cells activate surrounding cytotoxic T lymphocytes, helping the cytokines kill the tumor cells more effectively. In addition, Th2 and Th17 cells have different roles. Th2 cells induce the activation of B cells through humoral immunity but can reduce the Th1 immune response. Th17 cells promote the differentiation of fibroblast-like cells. Treg cells play an important role in reducing the immune response by depletion of IL-2, the expression of transforming growth factor beta and IL-10, and CTLA-4. Interestingly, it has been reported that O-GlcNAc is involved in the differentiation of Th cells. When TMG, a drug capable of inhibiting OGA, was administered to experimental animals, the levels of O-GlcNAc decreased [83]. Whether Th17 is a friend or foe in terms of tumor immune response is still unclear. In another study, O-GlcNAcylation was found to regulate Foxp3 [84]. Treg is well known as an important regulator of antitumor effect in tumor immunity. In Treg cells deficient in O-GlcNAc, it was confirmed that Foxp3 expression was reduced; and therefore, Treg functions were not effective, thus explaining the regulation of IL-2/STAT5 by O-GlcNAc. A recent study reported that STAT3 and the signal transducer and activator of transcription 5 (STAT5), which play important roles in Th17 cell differentiation and Treg differentiation, respectively, are important factors influencing T cell differentiation and can be regulated by O-GlcNAc [84,85]. In addition, O-GlcNAcylation of key signaling proteins that play an important role in T cells, such as nuclear factor of activated T cells (NFAT), affects the activation and function of T cells [86]. TCR in T cells acts as an important sensor that can detect and kill major histocompatibility complex molecules when presented with a malformed molecule such as cancer. TCR activation rapidly induced O-GlcNAcylation of NFATc1, and O-GlcNAcylated protein was observed in the nucleus within 5 min [86]. These results show that O-GlcNAcylation plays an important role in gene regulation of TF protein. This was verified through the reduction in TCR-induced production of IL-2 and activation markers such as CD69 through the inhibition of OGT [87].

### 4.3. Macrophage Differentiation and Activation by O-GlcNAcylation

Macrophages are derived from monocytes and play an important role in fighting an infection or the inflammatory response induced by pathogens [88]. In addition, macrophages perform an antigen presentation function through phagocytosis. Macrophages are myeloid-derived cells and are distinct from lymphocytes based on morphology and biological functions [89]. Macrophages in the tumor are divided into M1 and M2 macrophages (majorly M2-type macrophages). M1 macrophages express higher levels of IL-6, IL-10, and tumor necrosis factor alpha, thereby creating an immune suppressive environment. In contrast, M2 macrophages express higher levels of IL-10 and have a greater wound healing effect on the adjacent cells [90]. O-GlcNAcylation has been reported to affect the differentiation of M1 and M2 immune cells [85].

Several articles have reported that a normal level of OGT increases HBP, and that O-GlcNAcylation increases mitochondrial antiviral-signaling proteins (MAVS) to enhance innate immune response. O-GlcNAcylation plays an important role in attenuating infection with vesicular stomach virus [91]. O-GlcNAcylation enhances M1 macrophage polarization and inflammatory immune response [85]. In contrast, it was reported that the activity of HBP plays an important role in M2 macrophage differentiation. The N-glycosylation pathway plays an important role in activating CD206 and CD301, which are important markers of M2 macrophages in metabolic function [92]. Several studies have demonstrated that O-GlcNAcylation is involved in the activation of M1 and M2 macrophages. OGT increases the activity of lipopolysaccharide (LPS)-stimulated NF-κB and the expression of iNOS gene through mSin3a [93]. In addition, it has been confirmed in microglia cells, that c-Rel and p65 are regulated by O-GlcNAcylation. However, it has also been reported that strong O-GlcNAcylation affects the differentiation and activity of macrophages by inhibiting NF-κB p65 signaling [90]. This response is closely related to the response to the Toll-like receptor (TLR), which is an important function of macrophages. TLR4 signaling showed decreased activity by O-GlcNAcylation, revealing that O-GlcNAcylation is an important mechanism to regulate the innate immune response. LPS signaling increase due to lack of OGT is closely related to O-GlcNAcylation of RIPK3, which affects the phosphorylation of NF-κB and ERK. Phosphorylation of RIPK3 is enhanced in the absence of OGT, resulting in increased NF-κB and ERK signals. In addition, the activation of RIPK1, which affects necroptosis, is also associated with O-GlcNAcylation [94]. Activation of NOD2, which plays an important role in the innate immune response, promotes the expression and secretion of cytokines and chemokines. NOD2 is post-translationally modified by O-GlcNAcylation, and its stability and activity is mediated by O-GlcNAcylation [95].

### 4.4. O-GlcNAcylation and Tumor Microenvironment

Tumor microenvironment (TME) consists of various cells such as cancer cells, vascular endothelial cells, T cells, NK cells, macrophages, fibroblasts, and dendritic cells [96,97]. TME is a fairly complex system, including the metabolic interactions of various cells, and the direct interaction between cancer cells and immune cells. Macrophages and T cells are crucial in the TME [98,99]. Macrophages are involved in the role of tumor-antigen presentation, and immunosuppression in TME. Macrophages differentiate into M1- and M2-type macrophages, and occupy a high proportion in TME [97]. M1 macrophages are activated by stimuli such as external pathogens and interferon gamma, and have inflammation effects by secreting IL-12, thereby creating a tumor suppressive environment [100]. On the contrary, M2 macrophages are activated by IL-4, IL-10, and IL-13, and are known to have anti-inflammatory and immune suppressive effects on immune cells.

To overcome the immune suppressive TME created by M2 macrophages, various therapeutic agents are being developed to inhibit M2-type macrophage differentiation or increase the ratio of M1-type macrophages [101,102]. OGT may be a promising potential therapeutic target since OGT in macrophages is important in the regulation of phosphorylation of NF-kB, and the expression of iNOS and pro-inflammatory cytokine genes [93]. Further elucidation on the role of O-GlcNAcylation in M1 and M2 will help pave the way for OGT as a therapeutic target.

T cells also play a critical role in tumor suppression, and kill tumors by directly reacting with tumor antigens expressed on the surface of cancer cells (epitope-MHC complex) [103]. O-GlcNAcylation may function in the regulation of T-cell receptor (TCR) and T lymphocyte differentiation [104]. On the other hand, T cells inhibit the differentiation of immune cells, thereby reducing anti-tumor immune responses by OGT-induced T reg [84]. Thus, the dual mechanism of O-GlcNAcylation in T cells requires comprehensive immunological pre-clinical studies to define O-GlcNAcylation and its relationship with the anti-tumor effect in T cells.

## 5. Cancer Therapeutics Targeting O-GlcNAcylation

The role of hyper-O-GlcNAcylation in metastasis and resistance to chemotherapy in cancer as well as its potential role as a prognostic marker has prompted the development of targets directed at O-GlcNAcylation [17]. Cell lines of breast, colorectal, prostate, and hepatocellular carcinoma treated with investigational OGT inhibitors have shown a significant decrease in tumor growth [105,106,107,108,109]. Recently, tamoxifen-resistant breast cancer cell lines treated with OGT small molecule inhibitor OSMI-1 showed anti-tumor activity via epigenetic activation of the tumor-suppressor ERRFI1 [62]. Similarly, reductions in OGT levels have shown to inhibit growth of lung cancer cells; however, the role of OGT inhibitor in cancers have not been elucidated using an investigational agent [78].

Despite the promising pre-clinical data of OGT inhibitors, many hurdles remain, including the physiologic role of OGT involved in energy metabolism of normal cells [3]. Agents targeting OGT directly, such as small molecules and bisubstrate inhibitors, were initially ideal potential therapeutic options in the treatment of cancers [110]. However, the possibility of off-target toxicities for small molecules targeting OGT, and the inability to permeate through cells may render bisubstrate inhibitors ineffective [111].

## 6. Conclusions and Future Perspectives

In the past few decades, the mechanism of PTM of proteins has been extensively examined. Among the PTMs, including acetylation, ubiquitylation, and phosphorylation of proteins, the non-canonical glycosylation of O-GlcNAcylation is an emerging mechanism that remains to be fully elucidated. Cancer metabolism via O-GlcNAcylation is relatively unknown, when compared with other cancer hallmarks such as induction of angiogenesis, shift towards genome instability and mutation, sustenance of proliferative signaling, and avoidance of immune destruction.

O-GlcNAcylation plays a crucial role in the complex interplay of glucose metabolism in many diseases. The key concepts of the functions and mechanisms of O-GlcNAcylation in cancer cells have been recently elucidated. So far, we know that O-GlcNAcylation is associated with metastasis, interacts with RTKs, causes resistance to chemotherapy, may serve as a prognostic marker, and plays a role in immune surveillance in the tumor microenvironment. The complexity of O-GlcNAcylation currently hinders the comprehensive understanding of the mechanisms, which allows for proper selection and targeting of agents, such as OGT inhibitors, to exert anti-tumor activities. Further pre-clinical studies on O-GlcNAcylation will pave way for a better understanding of O-GlcNAcylation as a potential therapeutic target in many types of cancer.

## References

[1] Hanahan D., Weinberg R.A. (2011). Hallmarks of Cancer: The Next Generation. Cell.

[2] Fardini Y., Dehennaut V., Lefebvre T., Issad T. (2013). O-GlcNAcylation: A New Cancer Hallmark?. Front. Endocrinol..

[3] Liberti M.V., Locasale J.W. (2016). The Warburg Effect: How Does it Benefit Cancer Cells?. Trends Biochem. Sci..

[4] Marshall S., Bacote V., Traxinger R.R. (1991). Discovery of a metabolic pathway mediating glucose-induced desensitization of the glucose transport system. Role of hexosamine biosynthesis in the induction of insulin resistance. J. Biol. Chem..

[5] Bond M.R., Hanover J.A. (2015). A little sugar goes a long way: The cell biology of O-GlcNAc. J. Cell Biol..

[6] Hart G.W., Slawson C., Ramirez-Correa G., Lagerlof O. (2011). Cross talk between O-GlcNAcylation and phosphorylation: Roles in signaling, transcription, and chronic disease. Annu. Rev. Biochem..

[7] Torres C.R., Hart G.W. (1984). Topography and polypeptide distribution of terminal N-acetylglucosamine residues on the surfaces of intact lymphocytes. Evidence for O-linked GlcNAc. J. Biol. Chem..

[8] Hart G.W., Housley M.P., Slawson C. (2007). Cycling of O-linked β-N-acetylglucosamine on nucleocytoplasmic proteins. Nature.

[9] Yang X., Qian K. (2017). Protein O-GlcNAcylation: Emerging mechanisms and functions. Nat. Rev. Mol. Cell Biol..

[10] Hu P., Shimoji S., Hart G.W. (2010). Site-specific interplay between O-GlcNAcylation and phosphorylation in cellular regulation. FEBS Lett..

[11] Yi W., Clark P.M., Mason D.E., Keenan M.C., Hill C., Goddard W.A., Peters E.C., Driggers E.M., Hsieh-Wilson L.C. (2012). Phosphofructokinase 1 glycosylation regulates cell growth and metabolism. Science.

[12] Costa R., Remigante A., Civello D.A., Bernardinelli E., Szabó Z., Morabito R., Marino A., Sarikas A., Patsch W., Paulmichl M. (2020). O-GlcNAcylation Suppresses the Ion Current IClswell by Preventing the Binding of the Protein ICln to α-Integrin. Front. Cell Dev. Biol..

[13] Ruan H.-B., Singh J.P., Li M.-D., Wu J., Yang X. (2013). Cracking the O-GlcNAc code in metabolism. Trends Endocrinol. Metab. TEM.

[14] Bond M.R., Hanover J.A. (2013). O-GlcNAc cycling: A link between metabolism and chronic disease. Annu. Rev. Nutr..

[15] Zhu Y., Shan X., Yuzwa S.A., Vocadlo D.J. (2014). The Emerging Link between O-GlcNAc and Alzheimer Disease. J. Biol. Chem..

[16] Ferrer C.M., Sodi V.L., Reginato M.J. (2016). O-GlcNAcylation in Cancer Biology: Linking Metabolism and Signaling. J. Mol. Biol..

[17] Wu D., Jin J., Qiu Z., Liu D., Luo H. (2020). Functional Analysis of O-GlcNAcylation in Cancer Metastasis. Front. Oncol..

[18] Tan Z.-W., Fei G., Paulo J.A., Bellaousov S., Martin S.E.S., Duveau D.Y., Thomas C.J., Gygi S.P., Boutz P.L., Walker S. (2020). O-GlcNAc regulates gene expression by controlling detained intron splicing. Nucleic Acids Res..

[19] Love D.C., Kochran J., Cathey R.L., Shin S.-H., Hanover J.A. (2003). Mitochondrial and nucleocytoplasmic targeting of O-linked GlcNAc transferase. J. Cell Sci..

[20] Lazarus B.D., Love D.C., Hanover J.A. (2006). Recombinant O-GlcNAc transferase isoforms: Identification of O-GlcNAcase, yes tyrosine kinase, and tau as isoform-specific substrates. Glycobiology.

[21] Trapannone R., Mariappa D., Ferenbach A.T., van Aalten D.M.F. (2016). Nucleocytoplasmic human O-GlcNAc transferase is sufficient for O-GlcNAcylation of mitochondrial proteins. Biochem. J..

[22] Sacoman J.L., Dagda R.Y., Burnham-Marusich A.R., Dagda R.K., Berninsone P.M. (2017). Mitochondrial O-GlcNAc Transferase (mOGT) Regulates Mitochondrial Structure, Function, and Survival in HeLa Cells. J. Biol. Chem..

[23] Jóźwiak P., Ciesielski P., Zakrzewski P.K., Kozal K., Oracz J., Budryn G., Żyżelewicz D., Flament S., Vercoutter-Edouart A.S., Bray F. (2021). Mitochondrial O-GlcNAc Transferase Interacts with and Modifies Many Proteins and Its Up-Regulation Affects Mitochondrial Function and Cellular Energy Homeostasis. Cancers.

[24] Cheung W.D., Hart G.W. (2008). AMP-activated Protein Kinase and p38 MAPK Activate O-GlcNAcylation of Neuronal Proteins during Glucose Deprivation. J. Biol. Chem..

[25] Ruan H.-B., Han X., Li M.-D., Singh J.P., Qian K., Azarhoush S., Zhao L., Bennett A.M., Samuel V.T., Wu J. (2012). O-GlcNAc transferase/host cell factor C1 complex regulates gluconeogenesis by modulating PGC-1α stability. Cell Metab..

[26] Whisenhunt T.R., Yang X., Bowe D.B., Paterson A.J., Van Tine B.A., Kudlow J.E. (2006). Disrupting the enzyme complex regulating O-GlcNAcylation blocks signaling and development. Glycobiology.

[27] Lazarus M.B., Nam Y., Jiang J., Sliz P., Walker S. (2011). Structure of human O-GlcNAc transferase and its complex with a peptide substrate. Nature.

[28] Vucic D., Dixit V.M., Wertz I.E. (2011). Ubiquitylation in apoptosis: A post-translational modification at the edge of life and death. Nat. Rev. Mol. Cell Biol..

[29] Zachara N.E., O’Donnell N., Cheung W.D., Mercer J.J., Marth J.D., Hart G.W. (2004). Dynamic O-GlcNAc modification of nucleocytoplasmic proteins in response to stress. A survival response of mammalian cells. J. Biol. Chem..

[30] Ruan H.B., Nie Y., Yang X. (2013). Regulation of protein degradation by O-GlcNAcylation: Crosstalk with ubiquitination. Mol. Cell Proteom..

[31] Guinez C., Losfeld M.-E., Cacan R., Michalski J.-C., Lefebvre T. (2005). Modulation of HSP70 GlcNAc-directed lectin activity by glucose availability and utilization. Glycobiology.

[32] Guinez C., Mir A.-M., Leroy Y., Cacan R., Michalski J.-C., Lefebvre T. (2007). Hsp70-GlcNAc-binding activity is released by stress, proteasome inhibition, and protein misfolding. Biochem. Biophys. Res. Commun..

[33] Zhu Y., Liu T.-W., Cecioni S., Eskandari R., Zandberg W.F., Vocadlo D.J. (2015). O-GlcNAc occurs cotranslationally to stabilize nascent polypeptide chains. Nat. Chem. Biol..

[34] Hanover J.A., Krause M.W., Love D.C. (2012). Bittersweet memories: Linking metabolism to epigenetics through O-GlcNAcylation. Nat. Rev. Mol. Cell Biol..

[35] Li B., Li H., Lu L., Jiang J. (2017). Structures of human O-GlcNAcase and its complexes reveal a new substrate recognition mode. Nat. Struct. Mol. Biol..

[36] Elsen N.L., Patel S.B., Ford R.E., Hall D.L., Hess F., Kandula H., Kornienko M., Reid J., Selnick H., Shipman J.M. (2017). Insights into activity and inhibition from the crystal structure of human O-GlcNAcase. Nat. Chem. Biol..

[37] Roth C., Chan S., Offen W.A., Hemsworth G.R., Willems L.I., King D.T., Varghese V., Britton R., Vocadlo D.J., Davies G.J. (2017). Structural and functional insight into human O-GlcNAcase. Nat. Chem. Biol..

[38] Dennis R.J., Taylor E.J., Macauley M.S., Stubbs K.A., Turkenburg J.P., Hart S.J., Black G.N., Vocadlo D.J., Davies G.J. (2006). Structure and mechanism of a bacterial beta-glucosaminidase having O-GlcNAcase activity. Nat. Struct. Mol. Biol..

[39] Rao F.V., Dorfmueller H.C., Villa F., Allwood M., Eggleston I.M., van Aalten D.M.F. (2006). Structural insights into the mechanism and inhibition of eukaryotic O-GlcNAc hydrolysis. EMBO J..

[40] Schimpl M., Borodkin V.S., Gray L.J., van Aalten D.M. (2012). Synergy of peptide and sugar in O-GlcNAcase substrate recognition. Chem. Biol..

[41] Yang X., Su K., Roos M.D., Chang Q., Paterson A.J., Kudlow J.E. (2001). O-linkage of N-acetylglucosamine to Sp1 activation domain inhibits its transcriptional capability. Proc. Natl. Acad. Sci. USA.

[42] Housley M.P., Rodgers J.T., Udeshi N.D., Kelly T.J., Shabanowitz J., Hunt D.F., Puigserver P., Hart G.W. (2008). O-GlcNAc regulates FoxO activation in response to glucose. J. Biol. Chem..

[43] Lewis B.A., Hanover J.A. (2014). O-GlcNAc and the epigenetic regulation of gene expression. J. Biol. Chem..

[44] Singh J.P., Zhang K., Wu J., Yang X. (2015). O-GlcNAc signaling in cancer metabolism and epigenetics. Cancer Lett..

[45] Zhang K., Yin R., Yang X. (2014). O-GlcNAc: A Bittersweet Switch in Liver. Front. Endocrinol..

[46] Taylor R.P., Parker G.J., Hazel M.W., Soesanto Y., Fuller W., Yazzie M.J., McClain D.A. (2008). Glucose Deprivation Stimulates GlcNAc Modification of Proteins through Up-regulation of O-Linked-N-Acetylglucosaminyltransferase. J. Biol. Chem..

[47] Taylor R.P., Geisler T.S., Chambers J.H., McClain D.A. (2009). Up-regulation of O-GlcNAc transferase with glucose deprivation in HepG2 cells is mediated by decreased hexosamine pathway flux. J. Biol. Chem..

[48] Yang X., Ongusaha P.P., Miles P.D., Havstad J.C., Zhang F., So W.V., Kudlow J.E., Michell R.H., Olefsky J.M., Field S.J. (2008). Phosphoinositide signalling links O-GlcNAc transferase to insulin resistance. Nature.

[49] Kreppel L.K., Blomberg M.A., Hart G.W. (1997). Dynamic Glycosylation of Nuclear and Cytosolic Proteins: Cloning and characterization of a unique O-GlcNAc transferase with multiple tetratricopeptide repeats. J. Biol. Chem..

[50] Yang W.H., Park S.Y., Nam H.W., Kim D.H., Kang J.G., Kang E.S., Kim Y.S., Lee H.C., Kim K.S., Cho J.W. (2008). NFκB activation is associated with its O-GlcNAcylation state under hyperglycemic conditions. Proc. Natl. Acad. Sci. USA.

[51] Dehennaut V., Leprince D., Lefebvre T. (2014). O-GlcNAcylation, an Epigenetic Mark. Focus on the Histone Code, TET Family Proteins, and Polycomb Group Proteins. Front. Endocrinol..

[52] Whelan S.A., Dias W.B., Thiruneelakantapillai L., Lane M.D., Hart G.W. (2010). Regulation of insulin receptor substrate 1 (IRS-1)/AKT kinase-mediated insulin signaling by O-Linked beta-N-acetylglucosamine in 3T3-L1 adipocytes. J. Biol. Chem..

[53] Barkovskaya A., Prasmickaite L., Duveau D.Y., Mills I.G., Mælandsmo G.M., Moestue S.A., Itkonen H.M. (2017). Abstract 1131: O-GlcNAc transferase inhibition in breast cancer cells. Cancer Res..

[54] Akella N.M., Le Minh G., Ciraku L., Mukherjee A., Bacigalupa Z.A., Mukhopadhyay D., Sodi V.L., Reginato M.J. (2020). O-GlcNAc Transferase Regulates Cancer Stem–like Potential of Breast Cancer Cells. Mol. Cancer Res..

[55] Ma Z., Vosseller K. (2014). Cancer metabolism and elevated O-GlcNAc in oncogenic signaling. J. Biol. Chem..

[56] Chandrashekar D.S., Bashel B., Balasubramanya S.A.H., Creighton C.J., Ponce-Rodriguez I., Chakravarthi B., Varambally S. (2017). UALCAN: A Portal for Facilitating Tumor Subgroup Gene Expression and Survival Analyses. Neoplasia.

[57] Wieland E., Rodriguez-Vita J., Liebler S.S., Mogler C., Moll I., Herberich S.E., Espinet E., Herpel E., Menuchin A., Chang-Claude J. (2017). Endothelial Notch1 Activity Facilitates Metastasis. Cancer Cell.

[58] Ruland J. (2019). Colon Cancer: Epithelial Notch Signaling Recruits Neutrophils to Drive Metastasis. Cancer Cell.

[59] Lignitto L., LeBoeuf S.E., Homer H., Jiang S., Askenazi M., Karakousi T.R., Pass H.I., Bhutkar A.J., Tsirigos A., Ueberheide B. (2019). Nrf2 Activation Promotes Lung Cancer Metastasis by Inhibiting the Degradation of Bach1. Cell.

[60] Szymura S.J., Zaemes J.P., Allison D.F., Clift S.H., D’Innocenzi J.M., Gray L.G., McKenna B.D., Morris B.B., Bekiranov S., LeGallo R.D. (2019). NF-κB upregulates glutamine-fructose-6-phosphate transaminase 2 to promote migration in non-small cell lung cancer. Cell Commun. Signal..

[61] Carvalho-cruz P., Alisson-Silva F., Todeschini A.R., Dias W.B. (2018). Cellular glycosylation senses metabolic changes and modulates cell plasticity during epithelial to mesenchymal transition. Dev. Dyn..

[62] Zhang X., Sai B., Wang F., Wang L., Wang Y., Zheng L., Li G., Tang J., Xiang J. (2019). Hypoxic BMSC-derived exosomal miRNAs promote metastasis of lung cancer cells via STAT3-induced EMT. Mol. Cancer.

[63] Diepenbruck M., Christofori G. (2016). Epithelial-mesenchymal transition (EMT) and metastasis: Yes, no, maybe?. Curr. Opin. Cell Biol..

[64] Ali A., Kim S.H., Kim M.J., Choi M.Y., Kang S.S., Cho G.J., Kim Y.S., Choi J.-Y., Choi W.S. (2017). O-GlcNAcylation of NF-κB Promotes Lung Metastasis of Cervical Cancer Cells via Upregulation of CXCR4 Expression. Mol. Cells.

[65] Yan M., Xu Q., Zhang P., Zhou X.J., Zhang Z.Y., Chen W.T. (2010). Correlation of NF-kappaB signal pathway with tumor metastasis of human head and neck squamous cell carcinoma. BMC Cancer.

[66] Hu C.-M., Tien S.-C., Hsieh P.-K., Jeng Y.-M., Chang M.-C., Chang Y.-T., Chen Y.-J., Chen Y.-J., Lee E.Y.H.P., Lee W.-H. (2019). High Glucose Triggers Nucleotide Imbalance through O-GlcNAcylation of Key Enzymes and Induces KRAS Mutation in Pancreatic Cells. Cell Metab..

[67] Kaleem A., Ahmad I., Hoessli D.C., Walker-Nasir E., Saleem M., Shakoori A.R., Nasir ud D. (2009). Epidermal growth factor receptors: Function modulation by phosphorylation and glycosylation interplay. Mol. Biol. Rep..

[68] Waters A.M., Der C.J. (2018). KRAS: The Critical Driver and Therapeutic Target for Pancreatic Cancer. Cold Spring Harb. Perspect Med..

[69] da Cunha Santos G., Shepherd F.A., Tsao M.S. (2011). EGFR mutations and lung cancer. Annu. Rev. Pathol..

[70] Hsu W.H., Yang J.C., Mok T.S., Loong H.H. (2018). Overview of current systemic management of EGFR-mutant NSCLC. Ann. Oncol..

[71] Stateva S.R., Villalobo A. (2015). O-GlcNAcylation of the human epidermal growth factor receptor. Org. Biomol. Chem..

[72] Luanpitpong S., Angsutararux P., Samart P., Chanthra N., Chanvorachote P., Issaragrisil S. (2017). Hyper-O-GlcNAcylation induces cisplatin resistance via regulation of p53 and c-Myc in human lung carcinoma. Sci. Rep..

[73] Wang D., Wu J., Wang D., Huang X., Zhang N., Shi Y. (2021). Cisplatin enhances protein O-GlcNAcylation by altering the activity of OGT, OGA and AMPK in human non-small cell lung cancer cells. Int. J. Oncol..

[74] Rossi A., Maio M.D., Chiodini P., Rudd R.M., Okamoto H., Skarlos D.V., Früh M., Qian W., Tamura T., Samantas E. (2012). Carboplatin- or Cisplatin-Based Chemotherapy in First-Line Treatment of Small-Cell Lung Cancer: The COCIS Meta-Analysis of Individual Patient Data. J. Clin. Oncol..

[75] Dasari S., Tchounwou P.B. (2014). Cisplatin in cancer therapy: Molecular mechanisms of action. Eur. J. Pharmacol..

[76] Lee S.J., Kwon O.-S. (2020). O-GlcNAc Transferase Inhibitor Synergistically Enhances Doxorubicin-Induced Apoptosis in HepG2 Cells. Cancers.

[77] Itkonen H.M., Minner S., Guldvik I.J., Sandmann M.J., Tsourlakis M.C., Berge V., Svindland A., Schlomm T., Mills I.G. (2013). O-GlcNAc transferase integrates metabolic pathways to regulate the stability of c-MYC in human prostate cancer cells. Cancer Res..

[78] Mi W., Gu Y., Han C., Liu H., Fan Q., Zhang X., Cong Q., Yu W. (2011). O-GlcNAcylation is a novel regulator of lung and colon cancer malignancy. Biochim. Biophys. Acta.

[79] Lin Y.C., Lin C.H., Yeh Y.C., Ho H.L., Wu Y.C., Chen M.Y., Chou T.Y. (2018). High O-linked N-acetylglucosamine transferase expression predicts poor survival in patients with early stage lung adenocarcinoma. Oncotarget.

[80] Chiaradonna F., Ricciardiello F., Palorini R. (2018). The Nutrient-Sensing Hexosamine Biosynthetic Pathway as the Hub of Cancer Metabolic Rewiring. Cells.

[81] Qiang A., Slawson C., Fields P.E. (2021). The Role of O-GlcNAcylation in Immune Cell Activation. Front. Endocrinol..

[82] McClain D.A., Lubas W.A., Cooksey R.C., Hazel M., Parker G.J., Love D.C., Hanover J.A. (2002). Altered glycan-dependent signaling induces insulin resistance and hyperleptinemia. Proc. Natl. Acad. Sci. USA.

[83] Machacek M., Saunders H., Zhang Z., Tan E.P., Li J., Li T., Villar M.T., Artigues A., Lydic T., Cork G. (2019). Elevated O-GlcNAcylation enhances pro-inflammatory Th17 function by altering the intracellular lipid microenvironment. J. Biol. Chem..

[84] Liu B., Salgado O.C., Singh S., Hippen K.L., Maynard J.C., Burlingame A.L., Ball L.E., Blazar B.R., Farrar M.A., Hogquist K.A. (2019). The lineage stability and suppressive program of regulatory T cells require protein O-GlcNAcylation. Nat. Commun..

[85] Chang Y.H., Weng C.L., Lin K.I. (2020). O-GlcNAcylation and its role in the immune system. J. Biomed. Sci..

[86] Golks A., Tran T.T., Goetschy J.F., Guerini D. (2007). Requirement for O-linked N-acetylglucosaminyltransferase in lymphocytes activation. EMBO J..

[87] Swamy M., Pathak S., Grzes K.M., Damerow S., Sinclair L.V., van Aalten D.M., Cantrell D.A. (2016). Glucose and glutamine fuel protein O-GlcNAcylation to control T cell self-renewal and malignancy. Nat. Immunol..

[88] Hwang J.S., Kim K.H., Park J., Kim S.M., Cho H., Lee Y., Han I.O. (2019). Glucosamine improves survival in a mouse model of sepsis and attenuates sepsis-induced lung injury and inflammation. J. Biol Chem.

[89] Shapouri-Moghaddam A., Mohammadian S., Vazini H., Taghadosi M., Esmaeili S.A., Mardani F., Seifi B., Mohammadi A., Afshari J.T., Sahebkar A. (2018). Macrophage plasticity, polarization, and function in health and disease. J. Cell Physiol..

[90] He Y., Ma X., Li D., Hao J. (2017). Thiamet G mediates neuroprotection in experimental stroke by modulating microglia/macrophage polarization and inhibiting NF-κB p65 signaling. J. Cereb. Blood Flow Metab..

[91] Li T., Li X., Attri K.S., Liu C., Li L., Herring L.E., Asara J.M., Lei Y.L., Singh P.K., Gao C. (2018). O-GlcNAc Transferase Links Glucose Metabolism to MAVS-Mediated Antiviral Innate Immunity. Cell Host Microbe.

[92] Song N., Qi Q., Cao R., Qin B., Wang B., Wang Y., Zhao L., Li W., Du X., Liu F. (2019). MAVS O-GlcNAcylation Is Essential for Host Antiviral Immunity against Lethal RNA Viruses. Cell Rep..

[93] Allison D.F., Wamsley J.J., Kumar M., Li D., Gray L.G., Hart G.W., Jones D.R., Mayo M.W. (2012). Modification of RelA by O-linked N-acetylglucosamine links glucose metabolism to NF-κB acetylation and transcription. Proc. Natl. Acad. Sci. USA.

[94] Li X., Gong W., Wang H., Li T., Attri K.S., Lewis R.E., Kalil A.C., Bhinderwala F., Powers R., Yin G. (2019). O-GlcNAc Transferase Suppresses Inflammation and Necroptosis by Targeting Receptor-Interacting Serine/Threonine-Protein Kinase 3. Immunity.

[95] Hou C.-W., Mohanan V., Zachara N.E., Grimes C.L. (2015). Identification and biological consequences of the O-GlcNAc modification of the human innate immune receptor, Nod2. Glycobiology.

[96] Andrejeva G., Rathmell J.C. (2017). Similarities and Distinctions of Cancer and Immune Metabolism in Inflammation and Tumors. Cell Metab..

[97] Thorsson V., Gibbs D.L., Brown S.D., Wolf D., Bortone D.S., Ou Yang T.H., Porta-Pardo E., Gao G.F., Plaisier C.L., Eddy J.A. (2018). The Immune Landscape of Cancer. Immunity.

[98] Chanmee T., Ontong P., Konno K., Itano N. (2014). Tumor-Associated Macrophages as Major Players in the Tumor Microenvironment. Cancers.

[99] Zheng X., Mansouri S., Krager A., Grimminger F., Seeger W., Pullamsetti S.S., Wheelock C.E., Savai R. (2020). Metabolism in tumour-associated macrophages: A quid pro quo with the tumour microenvironment. Eur. Respir. Rev..

[100] Liu J., Geng X., Hou J., Wu G. (2021). New insights into M1/M2 macrophages: Key modulators in cancer progression. Cancer Cell Int..

[101] Cassetta L., Pollard J.W. (2018). Targeting macrophages: Therapeutic approaches in cancer. Nat. Rev. Drug Discov..

[102] Prenen H., Mazzone M. (2019). Tumor-associated macrophages: A short compendium. Cell Mol. Life Sci..

[103] Waldman A.D., Fritz J.M., Lenardo M.J. (2020). A guide to cancer immunotherapy: From T cell basic science to clinical practice. Nat. Rev. Immunol..

[104] Ramakrishnan P., Clark P.M., Mason D.E., Peters E.C., Hsieh-Wilson L.C., Baltimore D. (2013). Activation of the transcriptional function of the NF-κB protein c-Rel by O-GlcNAc glycosylation. Sci. Signal..

[105] Jiang M., Xu B., Li X., Shang Y., Chu Y., Wang W., Chen D., Wu N., Hu S., Zhang S. (2019). O-GlcNAcylation promotes colorectal cancer metastasis via the miR-101-O-GlcNAc/EZH2 regulatory feedback circuit. Oncogene.

[106] Itkonen H.M., Poulose N., Steele R.E., Martin S.E.S., Levine Z.G., Duveau D.Y., Carelli R., Singh R., Urbanucci A., Loda M. (2020). Inhibition of O-GlcNAc transferase renders prostate cancer cells dependent on CDK9. Mol. Cancer Res..

[107] Liu Y., Huang H., Cao Y., Wu Q., Li W., Zhang J. (2017). Suppression of OGT by microRNA24 reduces FOXA1 stability and prevents breast cancer cells invasion. Biochem. Biophys. Res. Commun..

[108] Itkonen H.M., Gorad S.S., Duveau D.Y., Martin S.E., Barkovskaya A., Bathen T.F., Moestue S.A., Mills I.G. (2016). Inhibition of O-GlcNAc transferase activity reprograms prostate cancer cell metabolism. Oncotarget.

[109] Xu W., Zhang X., Wu J.L., Fu L., Liu K., Liu D., Chen G.G., Lai P.B., Wong N., Yu J. (2017). O-GlcNAc transferase promotes fatty liver-associated liver cancer through inducing palmitic acid and activating endoplasmic reticulum stress. J. Hepatol..

[110] Trapannone R., Rafie K., van Aalten D.M. (2016). O-GlcNAc transferase inhibitors: Current tools and future challenges. Biochem. Soc. Trans..

[111] Borodkin V.S., Schimpl M., Gundogdu M., Rafie K., Dorfmueller H.C., Robinson D.A., van Aalten D.M.F. (2014). Bisubstrate UDP-peptide conjugates as human O-GlcNAc transferase inhibitors. Biochem. J..

